# A family-centered lifestyle intervention to improve body composition and bone mass in overweight and obese children 6 through 8 years: a randomized controlled trial study protocol

**DOI:** 10.1186/1471-2458-13-383

**Published:** 2013-04-25

**Authors:** Tamara R Cohen, Tom J Hazell, Catherine A Vanstone, Hugues Plourde, Celia J Rodd, Hope A Weiler

**Affiliations:** 1 School of Dietetics and Human Nutrition, McGill University, Montreal, QC H9X 3V9, Canada; 2 Department of Kinesiology and Physical Education, University of Lethbridge, Lethbridge, AB T1K 3M4, Canada; 3 Department of Pediatrics, McGill University, Montreal, QC H3A 1Y1, Canada; 4 Montreal Children’s Hospital, Montreal, QC H3H 1P3, Canada

**Keywords:** Childhood obesity, Lifestyle interventions, Body composition, Bone, Dairy, Family-centered

## Abstract

**Background:**

Childhood obesity gives rise to health complications including impaired musculoskeletal development that associates with increased risk of fractures. Prevention and treatment programs should focus on nutrition education, increasing physical activity (PA), reducing sedentary behaviours, and should monitor bone mass as a component of body composition. To ensure lifestyle changes are sustained in the home environment, programs need to be family-centered. To date, no study has reported on a family-centered lifestyle intervention for obese children that aims to not only ameliorate adiposity, but also support increases in bone and lean muscle mass. Furthermore, it is unknown if programs of such nature can also favorably change eating and activity behaviors. The aim of this study is to determine the effects of a 1 y family-centered lifestyle intervention, focused on both nutrient dense foods including increased intakes of milk and alternatives, plus total and weight-bearing PA, on body composition and bone mass in overweight or obese children.

**Methods/design:**

The study design is a randomized controlled trial for overweight or obese children (6–8 y). Participants are randomized to control, standard treatment (StTx) or modified treatment (ModTx). This study is family-centred and includes individualized counselling sessions on nutrition, PA and sedentary behaviors occurring 4 weeks after baseline for 5 months, then at the end of month 8. The control group receives counselling at the end of the study. All groups are measured at baseline and every 3 months for the primary outcome of changes in body mass index Z-scores. At each visit blood is drawn and children complete a researcher-administered behavior questionnaire and muscle function testing. Changes from baseline to 12 months in body fat (% and mass), waist circumference, lean body mass, bone (mineral content, mineral density, size and volumetric density), dietary intake, self-reported PA and sedentary behaviour are examined.

**Discussion:**

This family-centered theory-based study permits for biochemical and physiological assessments. This trial will assess the effectiveness of the intervention at changing lifestyle behaviours by decreasing adiposity while enhancing lean and bone mass. If successful, the intervention proposed offers new insights for the management or treatment of childhood obesity.

**Trial registration:**

ClinicalTrials.gov, NCT01290016.

## Background

Childhood obesity is a worldwide problem with immediate and long-term health consequences [1,2]. Not only are obese children at a greater risk of developing medical complications that include cardiovascular disease, type 2 diabetes, hypertension and lipid disorders [2], they are also at risk for developing low self esteem and often experience psychological distress [3]. Furthermore, it is reported that obese children may also present with orthopaedic and bone-related problems, including lower bone mass for their body weight [4,5], reduced bone strength [6] and increased risk of bone fractures [6,7].

Modifiable determinants of childhood obesity include energy intake (diet) and energy expenditure (physical activity (PA) and inactivity); these two components typically form the basis of prevention and treatment programs. Health Canada’s strategy to reduce childhood obesity addresses these two components, encouraging children to follow Canada’s Food Guide (CFG) [8] and participate in daily moderate to vigorous cardiovascular PA for 60-minutes per day [9]. Despite public health initiatives, statistics show that 1 in 5 Canadian children have energy intakes that exceed their energy expenditure [10]. Although children exceed their energy intakes, 71%, 37%, and 27% of children 4 to 8 y are not meeting the recommended servings of vegetables and fruit, milk and alternatives, and cereal products, respectively, per day [10]. Furthermore, only 9% of boys and 4% of Canadian girls accumulate 60 minutes of moderate-to-vigorous PA on at least 6 days a week, averaging 8.6 hours per day, or 62%, of their waking hours being sedentary [11].

Interventions involving overweight or obese children should address the modifiable behaviors of obesity together, including discussions about both diet and physical activity [12,13]. They should also be “family-centered” with at least one parent involved in the program [14]. The Transtheoretical Model (TTM) allows for assessment of participant awareness and acceptance of the obesity problem including their desires to change behaviors [15]. Applying TTM in programs can be accomplished through different interviewing techniques and counseling strategies in order to achieve individual goals [16]. Interventions should be sensitive to culture and socioeconomic status, as specific population groups may differ in terms of cultural needs and realistic lifestyle changes [17]. In addition, programs should to be tailored and include strategies such as goal setting [18], and educational sessions conducted by health professionals who deliver short and simple messages [19].

It is important that intervention programs start young, as health consequences can carry throughout adolescences to [1,2]. Programs targeting childhood obesity have been published using different study designs and interventions, making it is difficult to determine if the effects of the interventions favourably change body mass index (BMI) Z-scores and adiposity (fat mass) [20,21]. Musculoskeletal health of obese children has also been examined, showing that despite having higher bone mineral density (BMD) and bone size [4,5], obese children are at an increased risk of facture [22,23]. Studies assessing bone geometry and strength propose that the bones are not as “developed” and that the extra weight (fat mass) negatively effects bone [23]. The muscle-system is important for cortical bone health [24] and despite having elevated BMD, the increased weight on the cortical mass may be too high, thereby leading to increased fractures [22,23]. Furthermore, bone adapts to dynamic forces of muscle contractions, not by static forces of fat mass [25].

Research that has studied bone health in children uses different methodology techniques for assessing bone [22,25,26]. Recent studies that have use both dual-energy X-ray absorptiometry (DXA) and peripheral quantitative computed tomography (pQCT) allow for assessment of volumetric BMD (vBMD) (g/cm^3^) and bone geometry and not exclusively area BMD (aBMD) by DXA [22]. This has allowed for the discovery of negative relation to cortical and trabecular vBMD in obese children [27]. The relationship between bone and obesity also extends to environmental behaviors, including physical activity and nutrition [28]; research suggests that low circulating 25-hydroxyvitamin D (25(OH)D) concentrations [29-31] due to decreased sun exposure from sedentary lifestyles [32], poor diet [29], and increased vitamin D storage as it sequesters in adipose tissue [33,34]. Studies have attempted to enhance bone mass in children using dairy foods [35-37] or calcium supplements [38-41]. Doubling of calcium intake (from 750 mg/d to 1600 mg/d) in non-obese children enhanced bone density by 18.8% [42], suggesting a possible role for higher milk (calcium and vitamin D) intakes in improving bone health [43]. In addition to increased calcium intake, adding weight-bearing PA improves bone mass [44] and whole body bone mineral density (WB-BMD) [45].

Despite the well established role for calcium and milk in developing healthy bone mass, the contribution of milk and alternatives (i.e. cheese, yogurt, cottage cheese, soy beverages) and/or calcium achieving or maintaining healthy body weight is less clear [46-49]. Reviews that aim to conclude if increased dairy or calcium intakes affect body weight report mixed evidence [47,50]. To date no study has evaluated the effects of increased milk and alternatives plus total and weight-bearing PA on body composition and indices of bone health in prepubescent children, using a family-centered lifestyle intervention encompassing both education and behavioral modifying techniques. A study that assesses bone and body composition in addition to biomarkers of bone and obesity in overweight/ obese children who participate in a lifestyle intervention by both DXA and pQCT is warranted [27].

To our knowledge, there is no study that has examined the effects of a family-centered lifestyle intervention that investigated the altered bone structure [51] or modulators of bone health in addition to those that can reduce adiposity [52]. This paper shares a comprehensive approach that will be used in a randomized controlled trial of a 1-y lifestyle intervention in overweight and obese prepubescent children. This study will involve two levels of intervention: one focused on meeting recommended age-specific targets for nutrition and physical activity (standard intervention [StTx]) and a second focused on increased milk and alternatives consumption and weight-bearing activities (modified intervention [ModTx]) as a means to enhance support for bone mass. It is hypothesized that children receiving the ModTx will have a lower BMI Z-score, lower waist circumference and improved weight-for-age Z-score and increased bone mass and strength at 12-months compared to children in the StTx and control groups. Secondary objectives are to examine the effects of the intervention on changes of children’s eating behaviour throughout the intervention compared to control.

This study, the McGill Youth Lifestyle Intervention for Food and Exercise (MY LIFE Study), embraces the family-centered approach in a non-institutional setting. The primary focus of the MY LIFE Study is to create realistic nutrition and PA goals, while providing families with the necessary education and behavioral tools necessary to help the child meet their immediate and long-term goals of attaining a healthy lifestyle.

The study methods are in accordance with the CONSORT guidelines for reporting randomized trials [53]. Ethical approvals were obtained from McGill University Faculty of Medicine Institutional Review Board and Montreal Public School Boards. The MY LIFE Study is registered online at http://www.Clinicaltrials.gov (Trial # NCT01290016).

## Methods/design

### Participants

Eligible participants are healthy children 6–8 y, living in or near Montréal, Québec, who are overweight or obese according to the World Health Organization weight-for-height BMI cut-off criteria [54]. In addition to parental consent, participants provide written informed assent by reading and signing an assent form written at the grade 1 level. Participants must be either English or French. Only one eligible child per household is included in the study. Exclusion criteria includes: 1) known or suspected serious, chronic illness of childhood, such as cancer, Crohn’s disease, nephrotic syndrome, rheumatic conditions, and diabetes, etc., or those with disturbances in bone, vitamin D or mineral ion metabolism including rickets, osteomalacia, liver disease, renal disease, immobilization (complete or partial), current fractures, and disorders of the parathyroid gland; 2) use in the past 3 months, medications known to affect bone and/or mineral ion metabolism including all glucocorticoids, phosphate therapy or vitamin D analogues and any bisphosphonates; 3) severe anemia precluding blood sampling (previously diagnosed); 4) established diabetes mellitus (any type) and 5) hyperlipidemia ascribed to non-dietary causes.

### Recruitment strategy

Participants are recruited through public and private elementary schools (kindergarten through grade 3), primary healthcare organizations including physician referrals, word of mouth and local advertisements through newspaper, internet, radio commercials and postal mailings. Recruitment in schools included placing a bilingual study brochure in the children’s homework folders. Due to the sensitivity of the topic, all brochures are placed in envelopes addressed “To the Parents/ Pour les parents”. Interested participants contacted the research team by electronic mail or telephone. Participants are screened for eligibility by a researcher during the initial telephone call. If eligible, the family is scheduled for a baseline assessment within three weeks of the screening call at the research unit.

### Outcomes

The primary outcome of this study is change in BMI Z-score from baseline to 12 months. Secondary outcome measures include changes in body weight (kg), waist circumference (cm), % body fat (%), lean body mass (g), BMD (g) and bone mineral content (BMC: g), architecture of bone (radius and tibia) as well as bone strength. Changes in food intake, PA and average time spent engaging in sedentary activity (minutes/day) are also assessed. Other outcomes include changes in eating behaviour and child’s perceptions of healthy nutrition and physical activity behaviours.

### Randomization and blinding

Children are randomized by a computer-generated list using stratified block randomization of three per block to maintain balance. Two stratification factors are implemented: gender and BMI (overweight and obese). In order to maintain blinding for non-intervention related measurements, children are randomized to control, StTx or ModTx groups by the registered dietitian who conducts the interventions. Participants are informed of their group at the end of their baseline visit. All other research staff are blinded.

### Study protocol

All assessments take place at the same research unit every 3 months (Table 1). Appointments are scheduled at the convenience of the family including weekend and evenings.

**Table 1:** The MYLIFE study design: assessment and scheduling of baseline, follow-up visits and intervention sessions

	**Baseline**	**3-mo**			**6-mo**			**9-mo**			**12-mo**		
**Blood sampled **^**a**^	●	●			●			●			●		
**Anthropometry **^**b**^	●	●			●			●			●		
**Body composition assessment**													
**DXA **^**c**^													
Whole-body	●	●			●			●			●		
**Bone assessments**													
**DXA **^**d**^													
Whole-body	●	●			●			●			●		
Lumbar spine vertebrae 1-4 (AP and AP-Lateral)	●				●						●		
Total hip	●				●						●		
Forearm (1/3 distal radius)	●				●						●		
**pQCT **^**e**^													
Radius (%: 4, 66)	●				●						●		
Tibia (%: 4, 14, 38, 66)	●				●						●		
**Muscle function **^**f**^	●	●			●			●			●		
**Questionnaires**													
Family Health (FHQ)	●												
Child Health (CHQ)	●	●			●			●			●		
Child Intention (CIQ)	●	●			●			●			●		
**Baseline education **^**g**^	●	● ^**h**^			● ^**h**^			● ^**h**^			● ^**h**^		
**Interventions**													
**Lifestyle intervention**	**End of Mo**	**1**	**2**	**3**	**4**	**5**	**6**	**7**	**8**	**9**	**10**	**11**	**12**
**Intervention groups**		●	●	●	●	●			●				
**Control group **^**i**^													●

^a^ Includes: Glucose, HbA1C, insulin, calcium, CBC-profile, lipid-profile, AST/ ALT, C-reactive protein, Estradiol, LH.

^b^ Includes: Weight, height, BMI, waist circumference, blood pressure, pulse and skin pigmentation by spectrophotometer (CM-700d/600d, Konica Minolta, Ramsey, NJ, USA).

^c^ Body composition assessed by DXA to yield: total mass (g), fat mass (g), lean + BMC (g) % BF, Android/ Gynoid ratio.

^d^ DXA measures of bone to yield: bone area (cm^2^), BMC (g), BMD (g/cm^3^).

^e^ pQCT measures both bone content [i.e. cortical density (mg/cm^3^)] and geometry [i.e. cortical area (mm^2^)].

^f^ Force plate assessment: jumping (force, power), sit-to-stand (force, power) and balance.

^g^ Basic Education: Performed by a registered dietitian; a basic review of nutrition and PA guide.

^h^ A dietitian is always present to answer questions and deal with age changes if in control.

^i^ Control group receives the 6 lifestyle interventions after completion of 12 mo of study.

### Baseline

All participants arrive at the research unit fasted for 12-hours. Child assent and parent consent are obtained. Child’s weight and height are measured to confirm BMI criteria are met. All children are tested for normal blood glucose using a glucometer; those with blood glucose above ≥ 6.9 mmol/L are advised to seek subsequent medical assessment and are not enrolled in the study. Those with a normal blood glucose value (< 6.9 mmol/L) have a 9 ml blood sample taken. Parents complete a socio-demographic questionnaire and with a researcher, the child answers a series of questions from a study-specific questionnaire (*Child Intention Questionnaire* (CIQ)). All families receive a general nutrition and PA educational session by a registered dietitian. Parents are instructed how to complete a 3-day food diary; they are provided with a pre-paid envelope and asked to mail the diary once completed. The food diary will capture 3 non-consecutive days including one weekend day. At the end of the baseline visit, children choose from a ball, ball and racket or jumping rope, as a part of remuneration for their time with the dual purpose to encourage physical activity. Parents are told which group they are in and schedule their next visit.

### Follow-up visits

Children return to the clinic every 3-months for in the 12-h fasted state for blood sampling (Table 1). Parents complete a questionnaire about child’s eating behavior, physical activity level, and sun exposure. During these visits, blood tests, anthropometry, body composition, bone assessments, and muscle function tests are performed. The CIQ is also completed. A dietitian is present at all follow-up visits to ensure parents receive the same nutrition and physical activity advice throughout the study. We believe that families require continuous support and therefore having a dietitian present at all study visits helps reduce participant withdrawal, especially for the control group.

### Measurements

#### Biochemistry

Immediately after blood is drawn, plasma samples are spun for 20-minutes. Serum samples are spun after 30 min, and used to measure insulin (0.5 ml) while plasma (0.5 ml) is used for measurement of glucose, liver enzymes (ALT, AST) and lipids (LDL, HDL, total cholesterol, TG and hormones) using autoanalzyers (Beckman Access and Beckman DxC600 CA, USA) at the Montreal Children’s Hospital (MCH) clinical chemistry laboratory (certified by the provincial quality assurance program, the Laboratoire de santé publique du QC). These samples are sent for analysis to the MCH within 4-hours of blood sampling. A pediatrician reviews all biochemical data; abnormal results are addressed on an individual basis to ensure children are healthy. These biochemical outcomes are in line with the 2006 Canadian clinical practice guidelines on the management and prevention of obesity in adults and children [55].

In addition to the recommended biochemistry, we will also examine in our research unit the following in the view of bone health outcomes and satiety. Vitamin D status is examined using plasma 25(OH)D concentration, as well as osteocalcin and PTH concentrations using an autoanalyzer (Liaison, DiaSorin, Ontario, Canada). Plasma is reserved to measure 1,25-dihydroxyvitamin D (1,25(OH)_2_D) as needed using LC-MS/MS (Warnex Bioanalytical Services, Quebec, Canada). Blood samples to analyze satiety markers are prepared by adding inhibitors (20 uL AEBSF and 20 uL DPP-4) to one 2 mL tube. Satiety markers are measured at McGill University using multiplex assays and luminex technology (EMD Millipore Corporation, Billerica, MA, USA): grehlin (acylated and nonacylated), GLP-1, neuropeptide YY, adiponectin and leptin. For red blood cell analysis, methanol-H_2_O-BHT is added in equivalent volume to red blood cell volume and spun. All CV% are <5% and typically 1 to 2%. HOMA-IR is calculated as the product of the fasting insulin (IU/ml) and plasma glucose (mmol/L)/22.5 [56].

#### Anthropometry

Anthropometry is measured using standard practices: height is measured to the nearest 0.1 cm using a stadiometer (Seca 214, Hamburg, Germany) and weight is measured to the nearest 0.1 lb using a calibrated balance-beam scale (Detecto, Missouri, USA) in standardized children’s clothing (facility’s cotton shorts, T-shirt, socks). Weight is converted to kg and BMI is calculated (kg/m^2^) and Z-scores are derived using the World Health Organization’s ANTRHO software (version 3.2.2, January 2011, Switzerland, Europe). Waist circumference is measured to the nearest 0.1 cm at the umbilicus [57]. All values are expressed in absolute units with standard deviation scores using the data from the World Health Organization [58].

#### Health measures

During all visits, a registered nurse measures blood pressure using a standard sphygmomanometer (Trimline PyMaH Corp, Flemington, New Jersey, USA) and manually takes the child’s pulse. Pubertal stage is assessed by parent’s identification of physical development using the Tanner criteria [59,60]. Fasting serum concentrations of luteinizing hormone (LH) in boys, and LH and estradiol (E2) in girls is used to confirm Tanner stage.

### Body and bone assessment

#### Dual-energy X-ray absorptiometry (DXA)

DXA is used in the pediatric population to provide measurements of BMC and areal-BMD (aBMD) [61]. As per the International Society for Clinical Densitometry [61], whole body, lumbar spine vertebrae 1 to 4, total hip and forearm (non-dominant) BMC and aBMD are measured using a Hologic 4500A clinical densitometer (APEX version 13.2:3, Hologic QDR-4500A Discovery Series, Bedford, MA). For this test, children wear standardized clothing (cotton shorts, T-shirt, socks). Values are compared to the Hologic normative databases and thus expressed as absolute BMC and BMD along with Z-scores where applicable [61]. This Hologic model also measures soft tissue composition from the whole body scan, but does not distinguish subcutaneous from visceral fat depots. Information about android and gynoid regions, adipose indices (example: ratio of fat mass: height (kg/m^2^)) and lean plus BMC indices (example: [lean + BMC]/ height (kg/m^2^)) are also available and have been validated in children [62].

To complement the waist circumference measures, abdominal adiposity will be estimated using sub-region analysis of the full torso. Total body fat (kg and %) and trunk fat (kg) values are available from the DXA software. Although DXA measures of trunk fat mass reflect fatty elements in soft tissue as well as adipose tissue in subcutaneous and visceral depots, DXA-derived measures account for 80% of the variation in intra-abdominal fat as measured by computed tomography (CT) [4] and explain 79% of the variance in insulin sensitivity [63]. Values for total abdominal fat obtained using DXA are not different from CT measures [64]. Phantom scans are conducted daily to maintain quality assurance and the CV% for the phantom BMD and BMC will be calculated over the study period.

#### Peripheral quantitative computed tomography (pQCT)

To measure volumetric BMD (vBMD) as well as bone architecture, pQCT (XCT-2000; Stratec, Pforzheim, Germany) will be used [65]. This technique distinguishes the type of bone (cortical and trabecular) [66] in the non-dominant radius and tibia. Dominance will be established by asking the child which hand they write with; that side determines the dominant leg too. A 30-mm planar scout view is used to locate a standard anatomical site for the reference line at the distal end of the limb being measured. For the radius, length of the non-dominant forearm is measured as the distance between the olecranon and the styloid process forming the basis for the location of the distal and proximal slices. Single axis 2.5-mm slices (voxel size, 0.5 mm) are measured 4% and 66% proximally from the distal end of the radius. For the tibia, length of the non-dominant tibia is measured as the distance from the distance between the palpated lateral condyle and the lateral malleolus. Single slices are measured 4%, 14% 38% and 66% proximally from the distal end of the radius and tibia.

The scans are analyzed using contour mode 2 (45%) and peel mode 1 to assess total (TB) and trabecular bone (Trab) parameters at the 4% site. At the 66% site, cortical bone (Cort) is detected with separation mode 1 and a threshold of 710 mg/cm^3^. A similar method will be used for distal tibia. Normative data for adults not pediatrics exist for this model [67] and thus values will be tested for changes over time or as absolute changes. vBMD measurements are performed with a repeated-measures program that allows the starting point to be set according to previous measurements. Phantom scans are conducted daily to maintain quality assurance and the CV% for the phantom vBMD will be calculated over the study period. Data is analyzed using the manufacturer’s software package in which the outer contour of bone is defined with a threshold of 280 mg/cm^3^.

### Muscle function

At each research visit, balance and muscle strength are determined using standardized manoeuvres on a force plate (Model 9260AA, Kistler Instrument Corp., NY, USA). This force plate measures ground reaction forces, moments, and centre of pressure during balance testing using a piezoelectric 3-component force sensor in 3 planar directions. Manoeuvres include two-legged jumps, sit-to-stand exercises and a balance test. First, in 5-second increments, children are instructed to stand on the platform, bend their knees slightly and jump as high as they can with their hands freely beside them. They perform 6 repetitions. Secondly, seated with legs at a 90-degree angle and feet firmly placed on the platform, children “hug themselves” and rise out of the chair into a standing position (~4 seconds) and then sit back down in the chair back to the initial seating position for 4 seconds before repeating this exercise for 6 repetitions. To assess balance, children randomly close or open their eyes for three different sequences, 25 seconds per sequence. This exercise is repeated in random order. All data is analysed using Bioware Software (Kistler Instrument Corp.) and statistically used with DXA, pQCT, PA questionnaire and dietary data to assess for changes in muscle strength throughout the intervention.

### Demographics and home setting

At baseline only, parents complete a questionnaire that contains questions adapted from the Canadian Community Health Survey (CCHS) [68]. This questionnaire asks for parental age at birth of the index child, education, ethnicity, family income range and current employment. Neighbourhood environment is assessed by asking parents to report on proximity to outdoor and indoor recreational facilities, supermarkets and neighbourhood safety [69]. These questions are found in the study-created questionnaire titled the “Family Health Questionnaire” (FHQ).

### Parental perceptions of child obesity, intentions, nutrition knowledge and perceived behavioral control

Parents complete a set of questions about previous dieting and weight history, specifically how they perceive themselves as a child, adolescent and adult prior to birth of the participant [15]. They self-report current weight and height and report physical activity practices using the validated International Physical Activity Questionnaire [70]. Parents are also surveyed on their perceptions of their child’s weight using a visual analog scale [71]. Nutrition beliefs and practices regarding healthy snacks and milk are assessed using questions from a validated nutrition survey [72]. Parental intention and perceived behavioral control are addressed by asking parents: “*How committed are you to participating regularly in family physical activity over the next month*?” and “*If you were motivated, how confident are you that you could participate in regular family-based physical activity over the next month*?” Parents answer on a 7-point likert scale: “extremely uncommitted”; “very uncommitted”; “somewhat uncommitted”; “somewhat committed”; “committed”; “very committed” and “extremely committed” [73]. These questions form part of the FHQ.

### Daily physical activity

Parents report their child’s weekly physical activity by completing a modified Physical Activity Questionnaire – Older Children (PAQ-C) [74]. The PAQ-C is valid for 8 y old children (Canadian) and reflects the past week and captures activity in general, at school (physical education classes, recess, lunch), after school and weekends. This questionnaire does not include time and intensity and was therefore modified using the Canada Fitness Survey for children 7 y of age and older [75]. Modifications included using the duration and intensity format from the fitness survey and MET calculations. We also included “other” questions for PAQ-C to capture screen time including video gaming and surveying non-active or interactive involvement. The PAQ-C is part of our study questionnaire titled the “Child Health Questionnaire” (CHQ).

#### Validation of mPAQ-C

In view of our modified PAQ-C, 30 children (n = 10/group), are asked to complete a 7 day activity diary to serve as a mini-validation of our modified questionnaire. To reduce participate burden the 7-day diary will be voluntary at a convenient time. This validation will include a pedometer (StepCounts Co., Diabeters Inc., Deep River, ON) and accelerometer (ActiGraph GT3X, ActiGraph LLC, Fort Walton Beach, Florida). During a visit, children are shown how to wear both accelerometer and pedometer, how to conduct a 20- step test and how to reset the pedometer. Children are sent home with the pedometer, accelerometer, a pedometer log book, activity diary and a copy of the modified PAQ-C. Children wear the pedometer and accelerometer for 7 days and at night record the pedometer wear time. Each day, child and parent complete the activity diary reflecting the activity done on that day, including the intensity of activity. To validate the questionnaire, at the end of the week, parents complete the modified PAQ-C and mail all material back to the research unit.

Data analysis includes comparing pedometer log books to accelerometer readings. Activity diary, log book and accelerometer analysis is used to validate the modified PAQ-C by quantifying activity in terms of intensity (light, moderate or vigorous), type of activity and metabolic equivalents (METs).

### Dietary intake

Dietary intake is documented using 3-d food diaries. Parents complete the food diaries before each visit at baseline, 3, 6, 9 and 12 mo. These diaries reflect 2 weekdays and 1 weekend day and have been used previously in this age group [76]. Diaries are analyzed using Nutritionist Pro software (Axxya Systems, Stafford, TX), a program that uses the Canadian Nutrient File 2010. Data are analyzed according to CFG food groups, foods considered “extras”, macronutrients (carbohydrate, protein and fat), as well as micronutrients (calcium, vitamin D, sodium, potassium and iron). Differences in dietary intake are analysed throughout the study and considered as possible covariates in all statistical analyses.

### Exposure to UVB

To augment other information (outdoor activity, mobilization of fat stores or milk intake) as related to vitamin D status, exposure to sunshine, typical clothing habits, duration and frequency of exposure to sun, travel, and use of sunscreen are assessed using a questionnaire, which is found in the CHQ. To compliment this questionnaire, we measure skin pigmentation using a computerized narrow band reflectometer spectrophotometer (CM-700d/600d, Konica Minolta, Ramsey, NJ, USA). The spectrophotometer provides measures of melanin content of the skin or *Melanin Index* which has been shown to influence circulating 25(OH)D concentrations [77]. Measurements performed on the unexposed skin of the inner upper arm have higher correlation to the *Melanin Index* than measurements performed on the exposed forehead [78] according to standard guidelines [79]. At each visit, skin pigmentation established by measuring pigmentation three times at each site for constitutive pigmentation at the inner upper arm and facultative pigmentation at the forehead, mid-forearm and lower leg using the spectrophotometer. Individual typological angle (ITA^o^) is calculated with the L* and b* values using the equation from the Commission D’Éclairage [80] and are classified into 6 skin types based on Fitzpatrick descriptions [81,82].

### Parental perception of child eating behavior

Parents self-report on their child’s eating behaviour by completing the Child Eating Behaviour Questionnaire (CEB-Q) [83]. This validated questionnaire for children 4 to 13 requires parents to score 35 questions identifying different eating behaviours as “never”, “rarely”, “sometimes”, “often” or “always” [84-86]. This 2-pg survey identifies 7 eating styles: (1) food responsiveness; (2) enjoyment of food; (3) emotional overeating; (4) under eating; (5) desire to drink; (6) satiety responsiveness and slowness in eating; and (7) fussiness. The CEB-Q is completed at each research visit to help identify changes in child’s eating styles throughout the year and interventions. Unique to other studies, this questionnaire is used to individualization of the intervention sessions, by guiding the interventionist to discuss and prioritize negative eating behaviours. This questionnaire is part of our CHQ.

### Child intention and self-perception of nutrition and physical activity

A series of questions asked by a trained researcher to the child was created on the basis of the theory of planned behaviour [87]. Briefly, this theory postulates that human behaviour is guided by behavioral beliefs (e.g. consequences or attributes of the behavior), normative beliefs (e.g. expectations of others) and control beliefs (e.g. presence of factors that hinder or enhance behavior) [88]. These concepts are captured in the Child Intention Questionnaire (CIQ), a questionnaire that is research-administered every visit and asks children about their intentions and behaviours for engaging in physical activity, self-perception, attitude towards physical activity and nutrition and behavioural control [89,90]. Children self-identify themselves by identifying a picture that best describes them. The concept of self-perception is identified as it will influence decision making and ultimately the child’s actions [91]. Using a gender specific 5-point visual analogue scale, children identify how they feel about certain activities. Intensity of activity is determined using a gender specific Rated Perceived Exertion Scale [92]. With parental permission, this questionnaire is completed without the presence of the parents or family. This type of questionnaire is important to include in a study of this nature, as it identifies concepts that are important in weight interventions, especially when evaluating the family’s confidence in achieving a particular goal or changing a behavior. If interventions do not include such questions, it is likely goals will not be met and negative feelings towards the program may occur [91].

### Intervention

In line with the Transtheoretical Model [15] and the Theory of Planned Behavior [93], this study is based on similar principles of the “Obeldicks Light” framework [94]. Our intervention is based on physical activity, nutrition education and behavioral counselling. Interventions are family-centered and use motivational interviewing techniques, specifically engaging in reflective listening, sharing decision making with the child and families, and setting realistic goals with the child [95]. For example, the interventionist suggests goals to the child and as a group, all present discuss how the goals will be attained. A goal is never set unless the child agrees to the goal and can independently strategize how they can achieve the goal in a realistic manner. All members of the family are encouraged to participate in the interventions particularly those caregivers who have a direct impact on the child’s eating and activity (i.e. meal preparation, afterschool care etc.).

Intervention sessions are held at the end of each of the first 5 mo of the study, at the end of 8 mo, concluding with a debriefing session at 12 mo. Families participate in a total of 9 hours of counselling, based on 5 × 1.5 h visits at the end of months 1 through 5. The additional visit at the end of 8 months (1.5 h) is used to determine if new diet and activity intervention strategies are necessary over time as the child grows/matures and due to season. Counselling visits at the end of months 2, 5 and 8 overlap with regular study visits and are conducted after study data is collected to limit leading and bias (same day if need be to limit travel). Table 2 outlines the overall study design. All interventions have been designed by and will be carried out by a bilingual registered dietitian with experience in physical education.

**Table 2:** Differences among standard (StnInt) and modified (ModInt) treatment groups

** Standard intervention**	** Modified intervention**	**Control group**
**Nutrition recommendations **^**a**^		
Food servings per day^b^[9]:	Food servings per day^b^[9]:	Food servings per day:
•Vegetables and Fruit: 5	•Vegetables and Fruit: 5	•Will be based on the Canada Food Guide for their age.
•Grain Products: 4	•Grain Products: 4	
•Milk and Alternatives: 2	•Milk and Alternatives: 4	
•Meat and Alternatives: 1	•Meat and Alternatives: 1	
**Physical activity recommendations **^**a**^		
**Cardiovascular Recommendations**		
Frequency: 7 days	Frequency: 7 days	Frequency: 7 days
Intensity: moderate to vigorous	Intensity: moderate to vigorous	Intensity: moderate to vigorous
Type: cardiovascular	Type: cardiovascular	Type: cardiovascular
Time: 60 minutes	Time: 60 minutes	Time: 60 minutes
**Weight Bearing Recommendation**		
Frequency: 3 days	Frequency: 3 days	Frequency: 3 days
Intensity: light	Intensity: light	Intensity: light
Type: strength	Type: strength	Type: strength
Time: 30 minutes	Time: 30 minutes	Time: 30 minutes
**Sedentary activity**		
< 2 hours screen time per day	< 2 hours screen time per day	< 2 hours screen time per day

^a^ Children are encouraged to meet these recommendations for nutrition and physical activity. These recommendations are based on Canada’s Food Guide and the Canadian Physical Activity Guide. Children in the StnInt are reminded to engage in strength training activities, but the discussions are not as in depth as ModInt group.

^b^ If a child turns 9 their recommended to consume 6 Vegetables and Fruit; 5-6 Grains; 3 Milk and alternatives; 1 Meat and alternative.

The overall focus of the intervention is to help families make healthier lifestyle choices in terms of nutrition and physical activity, and to reduce screen time and other (sedentary behaviours). Differences between StTx and ModTx intervention lie in the CFG for children aged 4–8 y for all food group servings (Table 3). The modified intervention group is instructed to consume 4 servings of milk and alternatives per day. Although both interventions will be counselled to participate in 60-minutes of activity per day, special emphasis placed on activities that are also weight-bearing for ModTx. Examples of weight-bearing activities include jumping rope, brisk walking, dancing, racket sports (i.e. tennis) and soccer. Notably, these are also aerobic activities.

**Table 3:** **Intervention protocol for standard (StnInt) and modified (ModInt) treatment groups**^**a**^

**Visit 1**	**Visit 2**	**Visit 3**	** Visit 4**	** Visit 5**	** Visit 6**
**Education**					
1. Review CFG and PA guide^b^	1. Understanding Food labels^c^	1. Eating “out and about” (eating in other environments other than home)	1. Making a meal plan	1. Tricky situations	1. Staying on track
			2. Identifying hunger cues		
**Each session will also include discussions concerning:**					
•	Eating behaviours				
•	Reviewing the dairy intervention and strategizing how to stay on track				
•	Evaluating healthy food choices (Traffic light evaluation)				
•	Physical activity (frequency, type, time and intensity) discussion				
•	Sedentary activity (screen time) and provide alternative activities				
•	Relapse prevention through identification of a “tricky situation” (i.e. birthday party, vacation, holiday, sleepovers, rainy/ snowy days)				
•	Three SMART Goals				

CFGHE: Canada’s Food Guide to Healthy Eating, PA: Physical Activity.

^a^ Control group will receive the same visit format but at the end of 12-months of the study.

^b^ According to randomization, children are instructed to consume 2 or 4 servings of milk and milk alternatives per day.

^c^ Parents are encouraged to bring in food labels from home to ensure discussions are individualized.

All intervention sessions are recorded using a standardized tracking sheet for all components, ensuring all sessions follow the same structure with room for individualism and variety. Within each session, the dietitian identifies a key area that requires further discussion, which is either identified by evaluating the CEB-Q [83] or through parent concern or a concept that the interventionist has picked-up on during the session. This provides for individualization of sessions to meet family needs and to provide for self-directed change. All sessions are directed at the index child and are performed at the grade 1 reading and learning level.

### Components of the interventions

#### Education

The underlying principles of nutrition education are embedded in both StTx and ModTx groups. The goal of the education sessions is to improve self-efficacy through empowerment of healthy choices and behaviors. Table 2 details the education topics for each session. In brief, session 1 reviews the food and exercise guides. During session 2, families are taught how to read food labels and identify healthy food choices from labels using a tear-sheet from Health Canada [87]. Session 3 reviews eating out at restaurants or other environments. Session 4 asks children to create a meal plan using food models; during this session children are also taught about different types of hunger, a concept modeled from the Craving Change© series that focuses on three types of hunger (E.g. “tummy hunger”, “mouth and eye hunger” and “heart hunger”) [96]. Sessions 5 and 6 are open to reviewing concepts already discussed, and are necessary to discuss relapse prevention and reinforce positive lifestyle and behavioral changes. In line with TTM, sessions 5 and 6 are critical components of an intervention program as it ensures children and parents are able to remain in the action phase of change [15]. In addition, these sessions are used to increase self-efficacy of the child by discussing challenges that may arise and how to sustain positive thinking and motivation to stay on track.

#### Nutrition intervention and nutrition evaluation

According to the randomization, the dietitian counsels children to consume either 2 servings per day or 4 servings per day of milk and alternatives. Two servings per day is the recommendation for this age group (6–8 y). The child is given a certificate to remind parents of their specific milk and alternative requirements. Parents are also provided with the extended Health Canada list of milk and alternatives to ensure they understand this food group well. At this time, the dietitian works with the parents and child to strategize ways they can meet their recommendation. Ethically, and in line with Canada’s Food Guide, children who turn 9 will be instructed to consume 3 or 4 servings of milk and alternative as the recommendations suggest, but 3 per day accepted as meeting the servings.

At every intervention, parents and children complete a 24-h recall. Using the recall, the dietitian reinforces the appropriate quantity of milk and alternatives according to their group. If they are not meeting the recommendations, time is taken to discuss measures to get back on track and to identify barriers to change. Children are evaluated on their understanding of healthy snack choices using a modified traffic light diet evaluation, a form of evaluation often used with children [97]. The original traffic light diet uses a color-coded, calorie based exchange system [98]. We used the same concepts of coloring food choices and ask children to identify “go to foods” (green), “foods that are healthy but when eaten too much become unhealthy (yellow), and “foods that should be avoided on a routine basis, or “sometimes foods” (red). If necessary, the interventionist coaches the child by repeating the description of the food classification, but does not answer for the child. The interventionist records the answers and categorized by food group, or extra-foods (salty, sugary).

#### Physical activity intervention

Daily physical activity is discussed at every session. Children are asked if they remember the activity recommendations and how they can achieve it. Discussions are individualized per family using the FITT principle: frequency of activity, intensity, time and type [99]. Children are instructed to rate the intensity of their activities using a modified Borg Scale [92], which shows a child climbing stairs. Children rate their intensity by identifying the picture of the child on the stairs, with at the bottom (1) representing low intensity to the top with heavily sweating, red in the face and needing to stop (10) representing high intensity. At every session, children are encouraged to attain a 6–8 scale-rating (feeling sweaty, heart beating fast) for a total of 60-min of activity per day [9]. Using this scale not only allows researchers to understand child’s perceived intensity of activity, but also links to child’s self-efficacy. Different from the standard intervention, the modified intervention focuses on weight-bearing activities, such as jumping, running, or light strength training activities. These activities are recommended by the Canadian Society of Exercise Physiology and Health Canada and are monitored at every session [9].

#### Sedentary behaviour intervention

Time spent engaging in screen time is discussed during each intervention. Parents and children are taught that screen-time should be less than 2-h per day [100]; this includes computer gaming, telephone or internet time not related to schooling and television. Children are provided with a series of activities at each intervention session that will help them decrease their screen-time behaviours. They are asked to bring the completed activities to the next session for discussion. Specifically, session 1 asks children to record their total TV time for one-week using a calendar divided by 30-minute slots. At the end of each day, if they successfully watch less than 2-h per day, they are asked to color in a star. Session 2 children are encouraged to play a BINGO activity where they choose a different activity to play each day using a similar BINGO template. The time spent performing each activity is set by the child. Session 3 includes all family members as they try to complete a 1-month walking calendar where children color in feet (1 foot = 30 min of activity). All family members are encouraged to participate in this activity. Session 4 asks children to play a game based on a walk from their house to a castle, for every 30-min of moderate-intensity activity they may progress in the game by coloring in footstep towards the castle. Sessions 5 and 6 are open to provide children the activity they preferred the most and wish to complete, such as the walking calendar or BINGO. Should the child watch less than 2-hours of screen time prior to the intervention, sedentary activity is still discussed, monitored and positively reinforced.

#### Overcoming barriers: relapse prevention

Each visit includes a discussion with the dietitian about “tricky situations”; situations where one would either not follow CFG or have time to engage in PA. These situations may place children in a vulnerable situation where they know they will not be necessarily practicing healthy lifestyle choices, suggesting they may resort back to old habits of unhealthy lifestyles. Examples of tricky situations include birthdays, holidays, other celebrations, “sleep-over” parties, rainy days and vacations. With the help of the interventionist, realistic strategies and solutions are discussed to help child and family handle the situation to ensure they stay on track either on the day of or after the event.

#### Goal setting

Goal setting is a valid tool with this age group [91] and is a successful component of treatment programs. Goal setting with children provides insight to the child’s attitude and level of understanding of discussions during the intervention. Using a template adapted from Health Canada (Website: http://www.hc-sc.gc.ca/fn-an/food-guide-aliment/educ-comm/toolkit-trousse/plan-3a-eng.php), three SMART goals are set by the child. The goals must consist of at least one nutrition and one physical activity goal. The parents are present and can help suggest goals, but the child decides which goals they wish to pursue. The SMART goals consist of the following: *specific:* “What do I want to do?”; *measureable:* “How much and how often will I do it?” ; *attainable:* “How will I do it?”; *realistic:* “Can I do it?” and *time:* “When will I do it/ When will I start?”. The child signs the contract with the dietitian and is provided a copy for home. This is the final component of every intervention. Follow-up interventions always start with a review of goals. Children are reminded to be honest and to have open discussion as to why or why not the goals were attained in order to identify why some approaches work and barriers to others. Results from the interventions are recorded as: “yes, no or sometimes”. Creating realistic goals that are attainable and set by the participant is in line with clinical recommendations [55].

### Control

The control group visits the clinic every three months for the various assessments (Table 1). Ethically, appropriate care of the control group includes receiving the exact same number of counseling sessions at the end of the study. Specifically, after participating for one year, these families are offered the same number of counselling visits (Table 2). In terms of food group recommendations, specifically milk and alternatives, children will be instructed to follow the food guide appropriate for their age (i.e. 2–3 servings of milk and alternatives for 6–8 y olds and 3 to 4 servings of milk and alternatives for ≥ 9-y olds). Children are also encouraged to engage in 60 minutes of activity per day, including a variety of cardiovascular and strength training exercises. Frequency of these counselling sessions are determined by families.

### Sample size calculation

One-hundred and sixteen overweight/ obese children will be recruited to participate in this study. Based on similar studies [93-95], this sample size provides 80% power at a 5% significance level (two-sided) to detect a mean of change of BMI Z-score of −0.2 (SD 0.3). Estimating a 10% drop out rate, we aim to recruit 39 children for control, 39 children for StTx and 39 children for ModTx.

### Statistical analysis

All data is analyzed using SAS (Version 9.2, SAS Inc., Cary, NC). Summary statistics are computed for all baseline characteristics to ensure that the randomized treatment groups are different, and reported as 95% confidence intervals for each group. When baseline imbalances occur between groups despite randomization, these are treated as covariates and adjusted for. Mean absolute and change in BMI Z-score, waist circumference, lean, fat and bone mass, and biochemical indices are compared in each dose group under a mixed effects ANOVA model (similar to mixed effect regression as used in child obesity interventions [101]), with significant group differences localized by suitable post-hoc testing (e.g. Tukey method), again with adjustment for multiple comparisons to ensure a family-wise error rate of 0.05. Time course is evaluated under a mixed effects ANOVA model (with the addition of time as a random effect). Milk intake at baseline (0, 1 or 2 servings/d) may be used as a covariate. Relationships between outcomes are examined using correlation and regression analyses. For determinants of change in adiposity and bone mass, we explore age, sex, demographics, total energy intake, milk intake total activity or categories of inactive, activity etc., as possible “predictor” variables. During analysis of the data, when a mixed-effects regression model is deemed to give greater latitude in exploring the data we adopt that approach and also consider logistic regression.

## Discussion

This is the first family-based lifestyle intervention that targets prepubescent overweight and obese children with the intention to ameliorate both body composition and bone mass. The study focuses on the family as a whole, recognizing that both child and parents need to set attainable goals that focus on the child and indirectly require change at the home environment level. If effective, the tools and methods used in this study can be used in existing weight loss or child obesity treatment centers. A major strength of our study is our study design, that will determine if a program of such nature can support increases in lean mass, ameliorate impaired muscle function and increase bone density (areal and volumetric) as well as changes in adiposity (fat mass) over 1 y.

Our study is unique as it considers frameworks, such as behavioural and control beliefs of parent and self-identity of child [91]. From the child’s perspective, our study will be able to track changes in these beliefs and behaviours through research-administered questionnaires. This will help us understand the child’s intentions to change their behavior in order to sustain a lifestyle of healthy eating and regular physical activity. To date, our study is the first to develop a series of questions and track over 1-y changes in a child’s personal beliefs, self-perceptions and behaviours. Furthermore, we will be able to correlate these behavioral changes with changes in BMI Z-scores and changes in body composition from DXA. Unlike other studies, our intervention also includes a bone component, where we will be able to assess changes in dietary intake and physical activity as they relate to bone health, measured by DXA and pQCT.

The methods presented in this study build on previous work and findings in the area of child education, dietetic practice and nutritional assessments. Few studies have examined the effects of a long intervention of 1-y to reduce adiposity in children. This study is adequately powered to investigate the changes in both bone and body composition as they relate to the study group.

Nevertheless, counselling obese children poses unique challenges and barriers, including lack of overall family involvement, identifying participant motivation, support services, time and reimbursement [102]. Although including all family members in all sessions is important, it is true for some it may not benefit the child, particularly if parental level of support or willingness to change behaviour is not beneficial to the child [103]. A strength of the MY LIFE Study is the focus on the family environment and allows for sessions to be tailored to family needs. Our study offers continuous support to families by having dietitians available at all visits and conducting all follow-up visits at times that meet family needs. It is important that clinicians are capable of identifying behaviors that contribute to the child’s weight status and are able to work with the child to modify these behaviors [104]. The MY LIFE Study interventions are designed in a manner to support these needs, by using standard documentation techniques that utilize study tools (CEB-Q and PAQ-C) and still allow for variability of counselling topics. The MY LIFE Study provides an excellent example of how national educational tools, specifically the Canada’s Food Guide and Physical Activity Guide, can be used in a clinical setting. We believe that employing a family-centered approach that considers the family environment, developmental stage, as well as food and activity preferences will improve the program’s success. A focus on fun yet feasible activities will assist both children and parents in adhering to the program.

The results of the MY LIFE Study will be published in 2014 in relevant organizations and peer-reviewed academic journals. The study protocol presented in this paper is anticipated to help others who research the same area, and ultimately in creating treatment programs to combat childhood obesity.

## Abbreviations

PA: Physical activity; StTx: Standard treatment; ModTx: Modified treatment; CFG: Canada’s food guide; TTM: Transtheoretical model; BMI: Body mass index; BMD: Bone mineral density; DXA: Dual-energy x-ray absorptiometry; pQCT: Peripheral quantitative computed tomography; vBMD: Volumetric bone mineral density; aBMD: Area bone mineral density; MYLIFE: McGill youth lifestyle intervention for food and exercise; BMC: Bone mineral content; CIQ: Child intention questionnaire; FHQ: Family health questionnaire; PAQ-C: Physical activity questionnaire for children; CHQ: Child health questionnaire; CEB-Q: Child eating behavior questionnaire.

## Competing interests

Dr. Weiler received funding from the Dairy Farmers of Canada for this study. None of the other authors have a competing interest.

## Authors’ contributions

HW is the principle investigator of the study. HW, HP and CR designed the study. TC designed the interventions and study forms under the supervision of HW and HP. TC carries out all interventions and assists with all study visits, including DXA and muscle function assessments. TH conducts biochemistry, DXA, pQCT and muscle function assessments. CV takes bloods and conducts DXA, pQCT and muscle function assessments. All authors read and approved the final manuscript.

## Pre-publication history

The pre-publication history for this paper can be accessed here:

http://www.biomedcentral.com/1471-2458/13/383/prepub
